# Comparison of the hypotensor effect between latanoprost versus selective laser trabeculoplasty obtained with the water drinking test

**DOI:** 10.5935/0004-2749.20210052

**Published:** 2021

**Authors:** Renato Antunes Schiave Germano, Marcelo Hatanaka, Arthur Sônego Garcia, Flavio Augusto Schiave Germano, Caroline Schiave Germano, Felipe Biscegli Cid, Jorge Estéfano Germano

**Affiliations:** 1 Ophthalmology Department, Universidade de São Paulo, São Paulo, SP, Brazil; 2 Centro de Excelência em Oftalmologia, Bauru, SP, Brazil; 3 Ophthalmology Department, Irmandade da Santa Casa de Misericórdia de São Paulo, São Paulo, SP, Brazil

**Keywords:** Glaucoma, Intraocular pressure, Latanoprost, Lasers, Glaucoma, Pressão intraocular, Latanoprosta, Lasers

## Abstract

**Purpose:**

Glaucoma is the main cause of irreversible blindness worldwide. Peak intraocular pressure is one of the main risk factors for glaucoma progression, and intraocular pressure reduction remains the only therapeutic strategy for all types of glaucoma. The main purpose of our study was to compare the baseline and peak intraocular pressure reduction obtained with the water drinking test between the two eyes of the same patients using 0.005% latanoprost in one eye and selective laser trabeculoplasty application in the contralateral eye.

**Methods:**

This was a prospective, interventional, longitudinal, and randomized clinical trial, in which 30 consecutive glaucomatous patients, medically controlled using latanoprost monotherapy, were recruited from a single ophthalmological center. The patients’ eyes were randomized, and one eye was selected for SLT treatment and topical 0.005% latanoprost was introduced in the contralateral eye. The baseline intraocular pressure and peak intraocular pressure were evaluated 1 month (water drinking test 2) and 6 months (water drinking test 3) after treatment.

**Results:**

There was no significant difference between the mean pre-washout intraocular pressure in the randomized eyes for selective laser trabeculoplasty and latanoprost (13.6 ± 2.1 and 13.3 ± 1.8 mmHg, respectively; p=0.182). Regarding baseline intraocular pressure, there was no significant difference in the water drinking test 2 (p=0.689) and water drinking test 3 (p=0.06) between the groups. There was no significant difference in the intraocular pressure peak between the SLT and latanoprost groups at water drinking test 2 (p=0.771) or water drinking test 3 (p=0.774).

**Conclusions:**

The intraocular pressure reduction efficacy is similar between latanoprost and selective laser trabeculoplasty. Glaucomatous patients who are medically controlled with latanoprost and switch treatment to selective laser trabeculoplasty maintain control of intraocular pressure.

## Abstract (pt)

**Objetivo:**

Glaucoma é a principal causa de cegueira irreversível no mundo. O pico da pressão intraocular é um dos principais fatores de risco para progressão do glaucoma, e o controle pressórico ainda é o único tratamento efetivo para todas as formas de glaucoma. O objetivo principal deste estudo é comparar a redução basal e do pico da pressão intraocular, obtidas através do Teste de Sobrecarga Hídrica, entre os dois olhos dos mesmos pacientes utilizando latanoprosta 0,005% em um olho e submetidos à aplicação de trabeculoplastia a laser seletiva no olho contralateral.

**Métodos:**

Este é um estudo prospectivo, intervencionista, longitudinal e randomizado. Trinta pacientes consecutivos, glaucomatosos, com pressão intraocular controlada em uso de monoterapia com latanoprosta, foram recrutados de um único centro oftalmológico. Os olhos dos pacientes foram randomizados e um olho foi selecionado para tratamento com trabeculoplastia a laser seletiva e olho contralateral tratado com colírio de latanoprosta 0,005%. Foram avaliados a pressão intraocular basal e pico de pressão intraocular um mês (Teste de Sobrecarga Hídrica 2) e seis meses (Teste de Sobrecarga Hídrica 3) após tratamento.

**Resultados:**

Não houve diferença estatística entre a pressão intraocular pré washout entre os olhos randomizados para trabeculoplastia a laser seletiva e latanoprosta, 13,6 ± 2,1 e 13,3 ± 1,8 mmHg, respectivamente (p=0,182). Em relação à pressão intraocular basal, não houve diferença estatística entre os grupos, tanto no Teste de Sobrecarga Hídrica 2 (p=0,689) e Teste de Sobrecarga Hídrica 3 (p=0,06). Não houve diferença estatística em relação ao pico de pressão intraocular entre os grupos trabeculoplastia a laser seletiva e latanoprosta, no Teste de Sobrecarga Hídrica 2 (p=0,771) e Teste de Sobrecarga Hídrica 3 (p=0,774).

**Conclusões:**

Em resumo, nosso estudo demonsrou que a eficácia da redução pressórica é similar entre latanoprosta e trabeculoplastia a laser seletiva, e pacientes glaucomatosos que estão com a pressão intraocular clinicamente controlados com latanoprosta e trocam de tratamento para trabeculoplastia a laser seletiva mantém sua pressão intraocular controlada.

## INTRODUCTION

Glaucoma is the main cause of irreversible blindness worldwide^(1)^. It is a neuropathy characterized by the progressive loss of the ganglion cells of the retina and its axons, which presents as a specific lesion in the optic nerve, and a corresponding repercussion in the visual field^(2)^. Primary open-angle glaucoma (POAG) is an asymptomatic disease that requires rigorous investigation with optic nerve examinations, intraocular pressure (IOP) measurement, and visual field tests for diagnosis^(3)^. Nevertheless, estimates show that half of the cases are undiagnosed^(4)^.

The causes of POAG are multifactorial, and peak IOP is one of the main risk factors for glaucoma progression^(5,6)^. The water drinking test (WDT) was initially described by Schmidt^(7)^ for the diagnosis of glaucoma, but was later abandoned due to its low predictive capacity for the disease^(8)^. More recently, the WDT has been used as a tool to estimate unidentified IOP peaks during business hours, when IOP measurements are normally performed^(9,10)^. The pressure peaks that occur with the WDT allow estimation of the peak IOP that normally occurs during the day; this was demonstrated by a previous study that found a strong correlation between these peaks, and a concordance of ± 2 mmHg in 52.5% of exams^(9)^.

IOP reduction is the only therapeutic strategy for all types of glaucoma^(1)^. For the treatment of POAG, the first approach is usually the use of hypotensive drugs (beta blockers, alpha agonists, miotics, carbonic anhydrase inhibitors, prostaglandin analogues), while selective laser trabeculoplasty (SLT) has been used more recently^(11)^. Other therapeutic options include argonium laser trabeculoplasty (ALT), diode laser cycloablation, and surgical procedures such as trabeculectomy and artificial drainage tubes.

One of the first-line therapeutic drug options is 0.005% latanoprost, a highly selective synthetic prostaglandin F receptor drug, which has been shown to decrease IOP in patients with ocular hypertension, open-angle glaucoma, and normal-pressure glaucoma^(12)^. Latanoprost belongs to the class of prostaglandin analogues, which act to increase uveo-scleral drainage and is associated with a decrease in IOP of between 25% and 33%^(13)^. Latanoprost is a versatile drug, which is administered at night to maximize its effect during the day^(14)^. Moreover, topical latanoprost does not exceed the blood-aqueous barrier and, therefore, does not present hypotensive action in the contralateral eye^(15)^.

Selective laser trabeculoplasty is a technology that emits a 532 nm laser pulse, selectively applied to the pigmented trabecular meshwork; thus, it does not cause thermal damage in the non-pigmented trabecular meshwork^(16)^. Selective laser trabeculoplasty is advantageous because it leads to a moderate reduction in IOP with minimal side effects, besides the possible need to repeat the procedure^(17)^. Studies with SLT in 360^o^ of the trabecular meshwork showed a 20% and 30% reduction in IOP in 80% and 60% of patients, respectively, and when compared to latanoprost, there was no statistically significant difference in IOP reduction^(18)^.

The main purpose of our study was to compare the baseline IOP and peak IOP reduction between the two eyes of the same patients obtained with the introduction of 0.005% latanoprost in one eye and SLT application in the contralateral eye, using the WDT.

## METHODS

This is a prospective, interventional, longitudinal, and randomized clinical trial. The study was approved by the Ethics Committee of the institution. Thirty consecutive glaucomatous patients, medically controlled using latanoprost monotherapy in both eyes, were recruited from a single ophthalmological center.

Glaucoma was defined based on the presence of glaucomatous optic neuropathy (GON) and abnormal 24-2 SITA-Standard examinations (Humphrey Visual Field Analyzer; Carl Zeiss Meditec, Inc., Dublin, California, USA). GON was defined based on stereophotography evaluation by a glaucoma specialist using the following criteria: focal or diffuse neuroretinal rim thinning, focal or diffuse retinal nerve fiber layer loss, or inter-eye vertical cup-to-disc ratio asymmetry 0.2 not explained by differences in disc size. The visual field was determined to be abnormal if the glaucoma hemifield test (GHT) was out with normal limits and/or the pattern standard deviation (PSD) had a p-value <5% on at least two consecutive 24-2 examinations. The reliability indices were set at false-positive rates ≤10%, and false-negative rates and fixation losses ≤15%. Patients with significant lens opacity or ocular conditions that could affect visual field results were excluded. Only patients with open-angle glaucoma, defined during gonioscopic examination, were included. The inclusion criteria were patients >18 years old with POAG, who had medically controlled IOP lower than 21 mmHg with use of latanoprost in both eyes. The exclusion criteria were patients with angle closure or anterior peripheral synechia on gonioscopy, patients submitted to filtering surgeries, previously submitted to SLT, with unilateral or bilateral blindness, or who presented with decompensation of systemic diseases, such as diabetes mellitus or arterial hypertension.

After signing the informed consent term, all patients were required to stop using the drug in both eyes (wash out). After 15 days, patients attended a safety appointment for IOP measurement; if the IOP was >25 mmHg, the previous treatment was reinitialized and the patient was excluded from the study. After the 30-day wash out period, WDT 1 was performed, and treatment was introduced on the same day. Treatment was chosen according to an alternating assignment, as follows: The right eye of the first individual was selected for SLT treatment, then the left eye of the second individual, and so on, always alternating the eyes of the subsequent patients. Topical latanoprost (0.005%) was introduced in the contralateral eye, one drop, once a day, at night. All patients were asked to record their compliance in a daily diary.

The SLT laser used was the Ellex Solo (Ellex, Adelaide, Australia), q-switched Nd: YAG laser with the following characteristics: 532 nm emission, 3 ns pulse duration, double frequency, aim size of 400 µm, and pulse energies ranging from 0.8 mJ to 1.4 mJ, attached to a slit lamp.

All laser treatments were performed by a trained oph thalmologist. One drop of 0.4% oxybuprocaine eye drops was instilled in the eye before the procedure. A contact gonioscopy lens (Volk SLT Lens) was used, the laser focused and radiated the entire width of the trabecular meshwork. A single pulse was performed at the 12 o’clock position, initially set at 0.8 mJ. If no bubbles occurred, the energy was increased by 0.1 mJ until the appearance of the “bubbles of champagne” effect, indicating that the treatment power was adequate. The entire 360^o^ of the trabecular meshwork was treated with a total of 100 non-overlapping pulsations^(19)^.

After the procedure, non-steroidal anti-inflammatory eye drops (diclofenac) were prescribed three times a day for 3 days. No hypotensive eye drops were used.

An isolated IOP measurement was performed 1 hour after the procedure, and a 1-week safety return was scheduled for a new IOP measurement. The following visits were scheduled at 1 month, and then again between 4 and 6 months, in which WDT 2 and WDT 3 were performed, respectively.

For the WDT, all IOP measuments were performed between 2 pm and 4 pm. Patients were instructed not to ingest any liquid for at least 2 hours prior to the test. IOP was measured immediately prior to the ingestion of 800 ml of tap water in less than 5 minutes, and again 15, 30, and 45 min thereafter. The baseline IOP was the IOP measured before the ingestion of tap water, and the IOP peak was determined as the highest IOP measured during the WDT. IOP measurements were performed by two trained ophthalmologists using the same Goldman tonometer (Haag-Streit).

Statistical significance was defined at p<5% (α=0.05). The paired t-test and Student’s t-test were used to compare the effect of the treatment in each group with the basal IOPs. The covariance analysis (ANCOVA), with basal IOP as the covariate and treatment as the factor, was used to compare the pressure measurements during the WDT after the onset of treatment. Statistical analyses were performed with SPSS 11.0 software (SPSS, Inc., Chicago, IL, USA).

For a sample power of 80%, it was determined that a sample size of at least 28 eyes per treatment group was required to detect a difference of at least 1.5 mmHg between groups, assuming a standard deviation of 2.0 mmHg^(19)^, at a significance level of 0.05. Considering possible withdrawals during the study, we recruited a total of 30 patients.

## RESULTS

Thirty patients fulfilled our inclusion and exclusion criteria, but one patient did not attend for the WDT 3, and was excluded from the study. The included patients comprised 18 women (62%) and 11 men (38%), with a mean age of 56.6 ± 11.5 (34-75) years.

The right eye of 15 (51.7%) patients was designated to be submitted to SLT treatment, and the 14 (48.3%) remaining patients were treated with latanoprost. The contralateral eye of each group received latanoprost and SLT treatment, respectively.

There was no significant difference between the mean pre-washout IOP in the randomized eyes for SLT and latanoprost, 13.6 ± 2.1 and 13.3 ± 1.8 mmHg, respectively (p=0.182). Table 1 shows the results of the baseline IOP measurements (before the ingestion of water) between the two groups, before and after treatment. There was no significant difference in baseline IOP in WDT 1 (1 month after washout) between the groups (p=0.763). In the SLT group, treatment reduced the baseline IOP by 18%, in both the WDT 2 and WDT 3 (p<0.001). In the latanoprost group, there was a 19% reduction in baseline IOP in the WDT 2 (p<0.001), and 22% in the WDT 3 (p<0.001). There was no significant difference between the groups, in either the WDT 2 (p=0.689) and WDT 3 (p=0.06).

**Table 1:** Baseline IOP values (IOP before the ingestion of tap water) (± SD, mmHg)

	WDT 1 (basal, after washout)	WDT 2 (1 month)	WDT 3 (4-6 months)
SLT	16.6 ± 2.6	13.6 + 2.6 (p<0.001^*^ *vs* WDT1)	13.6 ± 2.2 (p<0.001^*^ *vs* WDT1)
Latanoprost	16.3 ± 2.5	13.2 ± 2.4 (p<0.001^*^ *vs* WDT1)	12.7 ± 2.0 (p<0.001^*^ *vs* WDT1)
Significance (SLT *vs* latanoprost)	p=0.763, *ns*	p=0.689, *ns*	p=0.06, *ns*

* Statistically significant.

*ns*= Not significant; SD= Standard deviation.

**Figure 1:**
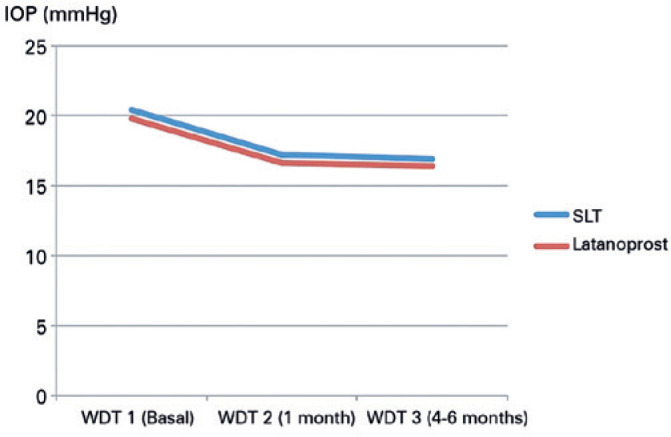
Peak IOP reduction during WDT, after 1 month (WDT 2), and after 4-6 months (WDT 3) compared to basal (WDT 1) in SLT and latanoprost groups.

Regarding the peak IOP obtained 1 month after washout with the WDT 1, there was no significant difference between the SLT and latanoprost groups (20.4 ± 3.3 and 19.8 ± 3.4 mmHg, respectively; p=0.06). In the SLT group, laser treatment reduced the peak IOP at WDT 2 and WDT 3 by 15.7% (p<0.001, versus WDT 1) and 17.2% (p<0.001, versus WDT 1), respectively. In the latanoprost group, clinical treatment reduced the peak IOP at WDT 2 and WDT 3 by 16.2% (p<0.001, versus WDT 1) and 17.2% (p<0.001%, versus WDT 1), respectively. There was no significant difference in IOP peak between the SLT and latanoprost groups at WDT2 (p=0.771) and WDT3 (p=0.774) (Table 2 and Figure 1).

**Table 2:** Peak IOP during the water drinking test (± SD, mmHg)

	**WDT1 (basal, after washout)**	**WDT2 (1 month)**	**WDT3 (4-6 months)**
SLT	20.4 ± 3.3	17.2 ± 3.9	16.9 ± 3.3
		(p<0.001^*^ *vs* WDT1)	(p<0.001^*^ *vs* WDT1)
Latanoprost	19.8 ± 3.4	16.6 ± 3.6	16.4 ± 3.3
		(p<0.001^*^ *vs* WDT1)	(p<0.001^*^ *vs* WDT1)
Significance (SLT *vs* latanoprost)	P=0.06	P=0.771, *ns*	P=0.774, *ns*

* Statistically significant.

*ns*= Not significant; SD= Standard deviation.

## DISCUSSION

Nagar et al.^(20)^ demonstrated better IOP control with topical use of latanoprost compared to SLT applied to 90^o^ or 180^o^ of the trabecular meshwork. In contrast, the application of 360^o^ SLT on the trabecular meshwork presented success rates and IOP reduction similar to the use of latanoprost, which led to the study of SLT as a primary treatment option for glaucoma. However, no previous studies have compared the efficacy of SLT versus latanoprost in decreasing IOP peaks obtained with the WDT.

SLT was first introduced in ophthalmology in 1995 as a new treatment to lower IOP levels^(16)^. The exact mechanism of action of SLT is not well established, and there are currently two main theories: The biological theory and the cell theory^(21,22)^. The biological theory proposes that laser energy causes a local wound, triggering a cascade of events that culminates with the attraction of macrophages that alter the secreted extracellular matrix, allowing an increased flow of aqueous humor. The cell theory, suggests that SLT applications stimulate cell division in the anterior trabecular meshwork, providing pluripotent cells for repopulation of the meshwork. These cells produce different extracellular matrices, increasing the output of aqueous humor. Regardless of the theory, the result of both mechanisms appears to be a reduction in outflow resistance, leading to improved outflow, and reduced IOP after SLT treatment^(21)^.

It has been suggested that the WDT could be used as an indirect predictor of the outflow facility reserve of the eye^(23)^. The acute intake of water elevates episcleral venous pressure, and may also cause choroidal engorgement, which may lead to increased resistance to aqueous outflow, causing a transient increase in IOP^(24)^. Treatment options that increase the aqueous humor outflow, such as SLT, would be expected to provide better IOP control during the WDT^(25)^. Vertrugno et al.^(26)^ performed the WDT in patients with POAG following treatment with different IOP-lowering medications to test the effect of drugs with different mechanisms of action on the ability to maintain a stable IOP. The authors concluded that topical medications that enhance outflow, such as latanoprost, may provide better IOP stabilization than those that decrease aqueous humor inflow. Our results showed that treatment with SLT and latanoprost achieved substantial IOP control, as both treatments significantly reduced the baseline and peak IOP during the WDT; these findings are in agreement with the results of previous investigations^(25,26)^.

In a recent multicenter randomized controlled trial published by Gazzard et al.^(27)^, hypotensive eye drops and SLT were compared as a first-line treatment for open angle glaucoma or ocular hypertension, in terms of health-related quality of life, cost, cost-effectiveness, clinical effectiveness, and safety. A total of 718 patients were enrolled in the study and were followed for 3 years. The results were favorable for SLT, and the authors demonstrated that the treatment with initial SLT is cost-effective with no significant difference in health-related quality of life and clinical outcomes, and lower cost compared with the conventional treatment with medication. They support a change in clinical practice, offering SLT as a first-line treatment for OAG and ocular hypertension.

In our study, glaucomatous patients who were initially medically controlled with latanoprost monotherapy and switched to treatment with SLT in one eye and maintained topical latanoprost in the contralateral eye showed a significant reduction in the baseline IOP and peak IOP measurements by the WDT in both eyes. There was no significant difference in efficacy between treatments with SLT or latanoprost. This information may be important in clinical practice because it is known that higher IOP peaks during the WDT are a predictive of future visual field progression^(28)^. Moreover, these patients may benefit from the advantages of SLT, such as the cost effectiveness of this treatment in comparison to hypotensive eye drops, as has been previously shown by Gazzard^(27)^.

Kerr et al.^(25)^ also studied the effect of SLT on peak IOP induced by the WDT, and showed a significant reduction in the mean baseline IOP, from 16.9 ± 2.4 to 14.2 ± 2.3 mmHg (p<0.001), as well as the peak IOP, from 21.9 ± 3.7 to 16.9 ± 3.1 mmHg (p<0.001). The results of this study are in agreement with those of the current study, in that we showed a mean baseline IOP decrease from 16.6 ± 2.6 to 13.6 ± 2.6 in the first month, and 13.6 ± 2.2 (p<0.001) after 6 months of laser treatment. Regarding the peak IOP, we found a significant reduction from 20.4 ± 3.3 to 17.2 ± 3.9 in the first month, and 16.9 ± 3.3 after 6 months of SLT (p<0.001).

In contrast to the previous literature, which mostly shows an IOP reduction equal or greater than 20%^(17)^, the IOP decrease in our study was less pronounced (<20%), both in the SLT and latanoprost groups. One possible explanation is that these patients did not have a very high initial IOP^(29)^, which may have decreased the efficacy of the treatment.

There are some limitations of our study. The time of latanoprost use before the study was not recorded, which made it impossible to analyze how this data could influence the results. In addition, the mean deviation values and the severity of the glaucomatous damage were not considered as inclusion or exclusion criteria; hence, it is not possible to verify whether the results could have been influenced by the disease severity. Another limitation is that the angle pigmentation was not quantified by the authors, which could explain the IOP reduction found in our study.

In summary, our study demonstrates that the IOP reduction efficacy is similar between latanoprost and SLT, and glaucomatous patients who are medically controlled with latanoprost and switch treatment to SLT maintain control of their IOP.
